# Detection of *Cronobacter* Genus in Powdered Infant Formula by Enzyme-linked Immunosorbent Assay Using Anti-*Cronobacter* Antibody

**DOI:** 10.3389/fmicb.2016.01124

**Published:** 2016-07-21

**Authors:** Xinjie Song, Shruti Shukla, Gibaek Lee, Sunhyun Park, Myunghee Kim

**Affiliations:** ^1^Department of Food Science and Technology, Yeungnam UniversityGyeongsan, South Korea; ^2^Food Standard Research Center, Korea Food Research InstituteSeongnam, South Korea

**Keywords:** *Cronobacter* species, immunoglobulin G, indirect non-competitive enzyme-linked immunosorbent assay, genus-specificity, powdered infant formula

## Abstract

*Cronobacter* species (*Cronobacter* spp.) are hazardous foodborne pathogens associated with baby food, powdered infant formula (PIF). To develop a rapid and sensitive method for simultaneous detection of seven *Cronobacter* spp. in PIF, an indirect non-competitive enzyme-linked immunosorbent assay (INC-ELISA) was developed based on a novel immunoglobulin G (IgG), anti-*Cronobacter* IgG. The developed INC-ELISA was able to detect seven *Cronobacter* spp. at concentrations ranging from (5.6 ± 0.30) × 10^3^ to (2.1 ± 0.01) × 10^5^ colony forming unit (CFU)/mL in pure culture. Further, INC-ELISA employing anti-*Cronobacter* IgG was applicable for analysis of PIF samples contaminated with less than <10 cells of *Cronobacter* spp. per 25 g of PIF in 36 h. The developed antibody showed slight cross-reactivity with *Franconibacter pulveris* (LMG 24057) at high concentration (10^8^ CFU/mL). The INC-ELISA method displayed excellent specificity without compromising cross-reactivity with other foodborne pathogens. The INC-ELISA assay method developed in this study using a novel anti-*Cronobacter* IgG facilitated highly sensitive, efficient, and rapid detection of *Cronobacter* spp. in baby food.

## Introduction

Bacteria of the genus *Enterobacter* are increasingly important human pathogens that cause neonatal meningitis and sepsis (Hunter and Bean, 2013). Enterobacterial infections have become a worldwide health problem partially due to the lack of development of preventive treatments and the emergence of new multi-drug resistant strains. *Enterobacter sakazakii* has recently been reclassified as a *Cronobacter* (Strydom et al., 2012). *Cronobacter* species (*Cronobacter* spp.), previously known as *E. sakazakii*, are foodborne pathogens that pose a high risk of infection to neonates as well as immuno-compromised individuals (World Health Organization [WHO], 2007). *Cronobacter* spp. affect the central nervous system of infants, and survivors often suffer from severe neurological impairments such as hydrocephalus, quadriplegia, and developmental delays (Lai, 2001; Strydom et al., 2012; Jaradat et al., 2014). Species level classification of the *Cronobacter* genus, which includes *C. sakazakii, C. muytjensii, C. malonaticus, C. turicensis, C. dublinensis, C. universalis*, and *C. condimenti*, was recently defined by Iversen et al. (2008) and Joseph et al. (2012b). *Cronobacter* spp. have been isolated from plant-based food products, including cereal, fruits, vegetables, legumes, herbs, and spices, as well as from animal-based food materials such as milk, meat, and fish (Friedemann, 2007; Lee et al., 2012).

All *Cronobacter* spp., except *C. condimenti*, have been associated with human infections (Cruz-Córdova et al., 2012). Historically, *Cronobacter* spp. have been implicated in newborn and infant infections, causing meningitis, necrotizing enterocolitis, and bacteremia (Healy et al., 2010). However, not all *Cronobacter* spp. are linked to infantile infections, and it is thought that virulence among strains may vary. *C. sakazakii, C. malonaticus*, and *C. turicensis* are most often isolated from infantile cases (Joseph and Forsythe, 2011). Recent research based on international microbiological standards suggested that all species of *Cronobacter* must be absent in 10 grams of powdered infant formula (PIF) (Odeyemi and Sani, 2016). For an understanding of recent unresolved issues persisting with respect to taxonomy, sources and clinical relevance, and for suggestions on how to safely feed premature neonates (Holy and Forsythe, 2014), it is plausible that virulence determinants have evolved in certain lineages (Joseph et al., 2012a).

Although reservoirs of *Cronobacter* spp. and their modes of transmission are still unknown, *Cronobacter* spp. have been suggested as a source of food contamination, with rodents and flies serving as a secondary route of contamination (Jung and Park, 2006; World Health Organization [WHO], 2007). According to a report by Jung and Park (2006), 20% of PIF samples were found contaminated with *Cronobacter* in the Republic of Korea. Lee et al. (2012) also reported similar results in which *Cronobacter* spp. were isolated from 18.6% of detected food samples. Although a number of reported cases of *Cronobacter* infection are quite low, sequelae can occur with high mortality rates (Lai, 2001; United States Food and Drug Administration [USFDA], 2002; Friedemann, 2007). World Health Organization [WHO] (2007), classified *Cronobacter* together with *Salmonella* as group A pathogens associated with PIF with clear evidence of illness in infants. These categories of organisms were based on their risk of illness to infants.

Culture-dependent isolation and assay methods for complete analysis of *Cronobacter* spp. from PIF usually require 5 to 7 days (United States Food and Drug Administration [USFDA], 2002). A further method was recommended by the International Organization for Standardization and the International Dairy Federation as ISO 22964 (Anonymous, 2006). This method includes a pre-enrichment in buffered peptone water (BPW), a selective enrichment in modified lauryl sulfate tryptose broth containing vancomycin and isolation of presumptive *C. sakazakii* colonies colored in blue-green on *E. sakazakii* isolation agar. These colonies should be streaked on tryptic soy agar and resulting yellow colonies are indicatory for *C. sakazakii*. However, this method was also time- and labor-consuming, as it usually requires 5 to 6 days to obtain a positive result (Norberg et al., 2012).

Lampel and Chen (2009) previously described a new method for the isolation and detection of *Cronobacter* spp. from PIF using a real-time PCR-based assay and chromogenic agar. In their study, suspended cells were isolated from enrichment culture, streaked onto chromogenic agar, and confirmed by real-time PCR assay. Mullane et al. (2006) also developed a method using cationic-magnetic beads to capture *Cronobacter* spp., and subsequent identification was performed after washing off bound cells from the capture phase and plating them onto Druggan–Forsythe–Iversen formulation agar to detect 1 to 5 colony forming unit (CFU)/500 g of PIF within 24 h. However, these PCR-based methods have significant technical requirements of ultra-pure reagents and chemicals along with high equipment costs. Therefore, a rapid, sensitive, and inexpensive method is needed for the detection of *Cronobacter* spp.

Polyclonal antibody can be obtained within a short time (4 to 8 weeks) with minimal financial investment, whereas it takes about 3 to 6 months to produce monoclonal antibodies (Leenaars and Hendriksen, 2005). Polyclonal antibody is commonly used in immunological methods, including enzyme-linked immunosorbent assay (ELISA), for the detection of foodborne pathogens (Brigmon et al., 1992; Kumar et al., 2008; Velusamy et al., 2010). It is also critical for developing a rapid and genus-specific method for identification of *Cronobacter* spp. in the context of food safety issues. Previously, a sandwich ELISA method was developed for the detection of *C. muytjensii* with a detection limit of 6.3 × 10^4^ CFU/mL using a species-specific anti-*C. muytjensii* immunoglobulin G (IgG) (Park et al., 2012). In addition, Xu et al. (2014) also developed a polyclonal and monoclonal antibodies-based indirect ELISA and a sandwich ELISA for the detection of *Cronobacter* spp. The indirect ELISA detected all species of *Cronobacter* assayed, and the limit of detection (LOD) was established as 10^5^ CFU/mL. In contrast, sandwich ELISA was specific for *C. sakazakii* and exhibited greater sensitivity than indirect ELISA (LOD of 2 × 10^4^ CFU/mL). Following 10 h of enrichment, *Cronobacter* spp. were detected using either of the two analytical methods in samples inoculated with 1 CFU/100 g PIF. The results from this study demonstrated that both of these novel ELISAs were specific, sensitive, and rapid assays for the screening of pathogenic *Cronobacter* spp. in PIF.

A widely available polyclonal antibody capable of detecting *Cronobacter* spp. could be used to avoid expensive and time-consuming methods. Thus, we decided to produce and characterize a polyclonal antibody for seven *Cronobacter* spp. To our knowledge, there has been no report on the ELISA-based detection of multiple *Cronobacter* spp. In the current work, an indirect non-competitive ELISA (INC-ELISA) method based on anti-*Cronobacter* IgG was developed in pure culture and applied for the detection of seven *Cronobacter* spp. in PIF.

## Materials and Methods

### Strains and Reagents

In the present study, seven *Cronobacter* strains were used to produce antibody and to assess the cross-reactivity of developed method, while eight other bacterial strains belonging to different genera were selected to check the cross-reactivity of developed assay due to their partial gene sequence similarity and to confirm the accurate detection of pathogen contaminants such as *Salmonella* spp., *Citrobacter* spp., and *Bacillus cereus* (Iversen et al., 2004; World Health Organization [WHO], 2007; Pinto et al., 2013). In the present study, *B. cereus* and *S.* Enteritidis were selected since it was found that the chances of contamination in PIF were highly concerned due to the presence of *B. cereus* and *S.* Enteritidis (Giammanco et al., 2011; Pinto et al., 2013). Giammanco et al. (2011) also reported that *Salmonella* spp. and *Cronobacter* spp. (formerly *E. sakazakii*) are the microorganisms of greatest concern in PIF. In addition, regarding the use of *Citrobacter freundii*, Giammanco et al. (2011) observed that molecular epidemiological survey of *C. freundii* was misidentified as *Cronobacter* spp. isolated from PIF. The phylogenetic tree also showed that *C. freundii* and *Cronobacter* spp. are very closely related species based on their smaller amounts of mahalanobis distances than other species. Hence, it was suggested that *C. freundii* may also be an under-reported cause of bacterial infection, especially in high risk neonates, due to misidentification. *B. cereus* (KCCM 40935), *Buttiauxella noackiae* (ATCC 51713), *C. condimenti* (LMG 26250), *C. dublinensis* (LMG 23823), *C. malonaticus* (LMG 23826), *C. muytjensii* (CDC 3523-75), *C. turicensis* (LMG 23827), *C. muytjensii* (ATCC 51329), *C. sakazakii* (ATCC 29544), *C. sakazakii* (ATCC 29004), *C. universalis* (LMG 26249), *C. freundi* (ATCC 8090), *Escherichia coli* (ATCC 39418), *Franconibacter helveticus* (LMG 23732), *Franconibacter pulveris* (LMG 24057), and *Salmonella* Typhimurium (ATCC 13311) were used in this study. *C. muytjensii* (CDC 3523–75) was donated by Dr. Carol Iversen from University College Dublin, Ireland. Other strains used in this study were purchased from the American Type Culture Collection (ATCC; Manassas, VA, USA) and Korean Culture Center of Microorganisms (KCCM; Seoul, Korea). Strains indicated with LMG were purchased from the Belgian Coordinated Collections of Microorganisms (BCCM; Gent, Belgium). All strains were cultured in nutrient broth (NB) for 18 h at 37°C in a shaking incubator (150 rpm).

NB, nutrient agar (NA), peptone, and skim milk were purchased from Difco (Franklin Lakes, NJ, USA). *Enterobacteriaceae* enrichment (EE) broth and violet red bile glucose (VRBG) agar were purchased from MB cell (Seoul, Korea). Sodium carbonate, sodium bicarbonate, sodium azide, potassium phosphate monobasic, potassium phosphate dibasic, sodium chloride, caprylic acid, ammonium sulfate, and alkaline phosphatase yellow liquid substrate *p-*nitrophenyl phosphate (pNPP) were purchased from Sigma (St. Louis, MO, USA). Phosphatase-labeled goat anti-rabbit IgG was purchased from Kierkegaard & Perry Laboratories, Inc. (Gaithersburg, MD, USA). Lipopolysaccharide (LPS) extraction kit was purchased from Intron Biotechnology (Seongnam, Korea).

### Animal Care Ethics

Animal use protocol was reviewed by the committee members of Yeungnam University and approved by Korea Food and Drug Administration, Republic of Korea (*Animal Ethics License No. 2013-012 and 2012-010*).

### Preparation of Immunogen and Immunization

Three types of immunogens were prepared for the development of a genus-specific antibody against *Cronobacter* spp. A sonicated cell protein (SCP) mixture of seven *Cronobacter* spp. was used in this study. The seven *Cronobacter* spp. were separately cultured in NB at 37°C for 18 h to a concentration of 1 × 10^9^ CFU/mL. Each culture (30 mL) was centrifuged at 3,000 × *g* at 4°C for 30 min. The pellets were washed three times with 30 mL of 0.01 M phosphate-buffered saline (PBS) and then suspended in 10 mL of 0.01 M PBS for sonication under 20 KHz power for 5 min on ice. Supernatant was collected after centrifugation at 12,000 × *g*, 4°C for 20 min. Protein concentrations of supernatants were checked with a Bradford kit, after which SCP solutions were adjusted to a concentration of 0.5 mg/L with 0.01 M PBS. SCP solutions were stored at -20°C before use. For immunization, equal volumes of the seven SCP solutions were mixed for use as an immunogen in New Zealand white rabbits.

LPS mixture of the seven *Cronobacter* spp. was prepared using an LPS extraction kit following the instructions of the manufacturer. Each *Cronobacter* spp. culture was centrifuged at 10,000 × *g* at room temperature for 20 min to harvest bacterial cells. Lysis buffer was then added and vortexed vigorously, followed by addition of chloroform, vortexing for 20 s, and incubated at room temperature for 5 min. The mixture was then centrifuged at 10,000 × *g* for 10 min at 4°C. An aliquot of supernatant was transferred to a new tube, mixed well with purification buffer, and kept at -20°C for 10 min. The solution was then centrifuged at 10,000 × *g* for 15 min at 4°C, after which the upper layer was removed to obtain an LPS pellet. The pellet was washed three times with 1 mL of ethanol, centrifuged for 3 min at 10,000 × *g* at 4°C, and then collected and dried at room temperature. For the LPS solution, 50 μL of Tris-HCl buffer (10 mM, pH 8.0) was used to dissolve the LPS pellet. LPS solution of the seven *Cronobacter* spp. was prepared by mixing an equal volume of each LPS solution for use as an immunogen in New Zealand white rabbits.

Formalin-killed cell (FKC) mixture was also prepared as an immunogen. Cultures of the seven *Cronobacter* spp. were applied as treatments with 0.5% formalin for 24 h, followed by centrifugation (3,000 × *g*, 4°C, for 30 min) according to the method of Song and Kim (2013). First injection was administered with a mixture of immunogen and Freund’s complete adjuvant (1:1, v/v). Second and third injections were administered using the same mixture of immunogen and Freund’s incomplete adjuvant (1:1, v/v) at 4 weeks after the first injection. Immunogen was injected into the back of rabbits at four sites at a concentration of 0.25 mL/site. Blood samples were taken every week until 18 weeks after the first injection.

### Preparation and Purification of IgG

Blood samples were centrifuged (10,000 × *g*) for 30 min at 4°C to separate the anti-sera. Anti-*Cronobacter* IgG was purified from the anti-sera by caprylic acid and ammonium sulfate precipitation as described by McKinney and Parkinson (1987) and according to further modified methods of Shukla et al. (2012) and Song and Kim (2013).

### Sodium Dodecyl Sulfate-Polyacrylamide Gel Electrophoresis (SDS-PAGE) for Purified Anti-*Cronobacter* IgG

The purity of various anti-*Cronobacter* IgGs preparations was checked using SDS-PAGE (Atto, Tokyo, Japan) under reduced conditions as described by Park et al. (2012) with some modifications.

### Development of INC-ELISA for Detection of *Cronobacter* spp.

Rabbit anti-*Cronobacter* IgG was used to develop an INC-ELISA method for detection of *Cronobacter* spp. Standard response curve of the seven *Cronobacter* spp. was constructed using the following procedure. First, fresh cultures of all tested *Cronobacter* spp. were diluted decimally with 0.05 M carbonate buffer (pH 9.5) in order to coat 96-well plates (SPL Life Sciences, Gyeonggi-do, Korea), whereas only carbonate buffer was used as a negative control. Simultaneously, coated cultures of *Cronobacter* spp. were counted on NA and VRBG agar plates for determination of concentration. After coating at 4°C overnight, 96-well plates were washed three times with 0.01 M PBS (pH 7.0) and blocked with 200 μL of 5% skim milk at 37°C for 2 h. Ninety six-well plates were then washed with 0.01 mol/L PBS-0.05% Tween 20 (PBST), added with rabbit anti-*Cronobacter* IgG, and incubated at 37°C for 1 h. Then, plates were washed again with 0.01 M PBST, after which 100 μL of phosphatase-labeled goat anti-rabbit IgG was added and the plates incubated at 37°C for 1 h. Plates were washed again with 0.01 M PBST, after which 50 μL of pNPP liquid substrate was added to each well for a 30 min enzyme-substrate reaction. Finally, 50 μL of 0.01 M NaOH solution was added to stop the reaction, after which the final reaction mixture was analyzed using an Infinite M200 (Tecan; Seestrasse, Switzerland). A detection limit of the developed INC-ELISA was determined by using a data linearity function and standard deviation of responses for the calibration standard and samples, respectively (Hubaux and Vos, 1970).

### Specificity and Sensitivity of INC-ELISA Assay

Seven common *Cronobacter* foodborne pathogens (**Table 1**) were used for the specificity test of the developed INC-ELISA. Cultures of tested foodborne pathogens were serially diluted to final concentrations of 10^0^ to 10^8^ CFU/mL and then used to check sensitivity of the developed INC-ELISA. All experiments were repeated three times.

**Table 1:** Detection of *Cronobacter* species in pure culture using the developed indirect non-competitive enzyme-linked immunosorbent assay.

Species	Results	Detection limits		Positive/Negative values
		CFU/mL	Log CFU/mL	
*Cronobacter condimenti*	+	(2.3 ± 0.08) × 10^4^	4.36	18.9
*Cronobacter dublinensis*	+	(5.7 ± 0.15) × 10^4^	4.75	10.9
*Cronobacter malonaticus*	+	(4.9 ± 0.24) × 10^4^	4.69	10.8
*Cronobacter muytjensii* (ATCC 51329)	+	(1.0 ± 0.05) × 10^4^	4	13.9
*Cronobacter sakazakii* (ATCC 29544)	+	(5.6 ± 0.30) × 10^3^	3.74	12.68
*Cronobacter turicensis*	+	(2.9 ± 0.16) × 10^4^	4.46	14.7
*Cronobacter universalis*	+	(2.1 ± 0.01) × 10^5^	5.32	6.0

### Food Trial Using *Cronobacter*-Spiked PIF Sample (Artificial Inoculation of *Cronobacter* into PIF Sample)

Firstly, the seven *Cronobacter* strains were separately cultured in NB at 37°C for 20 h, after which the cultures were serially diluted with BPW to obtain the lowest final concentration (<10 CFU/mL) of *Cronobacter* live cells for use in further experiments. To examine the applicability of the developed anti-*Cronobacter* IgG against each *Cronobacter* strain, 25 g of PIF sample was aseptically placed into a flask, mixed with 225 mL of BPW as a pre-enriched broth, and separately spiked with 1 mL (less than 10 cells/25 g of PIF) of each *Cronobacter* species, followed by pre-enrichment incubation at 37°C for 8 h. Following this, 1 mL of the pre-enrichment culture was inoculated into EE broth and incubated for 8 h of enrichment (Korea Food and Drug Administration [KFDA], 2014). Further, enriched aliquots of pre-treated food samples were tested using the developed INC-ELISA and standard USFDA methods. For control, non-spiked PIF samples were analyzed using the developed INC-ELISA and standard microbiological USFDA methods (United States Food and Drug Administration [USFDA], 2002). All experiments were performed in three replicates in order to maintain reliability of the work.

### Statistical Analysis

The detection limit was calculated as the average value of absorbance at zero concentration with three standard deviations (Park et al., 2012). All experiments and results were analyzed in at least three trials for proving reliable and reproducible data.

## Results

### Characteristics of Developed Antibodies

**FIGURE 1:**
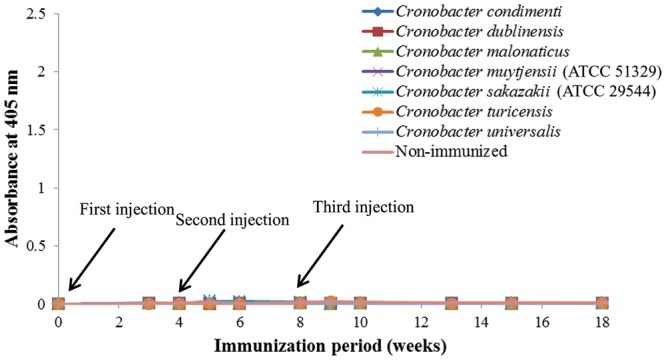
**Titers of rabbit anti-serum developed by lipopolysaccharide against seven *Cronobacter* species.** All experiments were conducted three times, and data represent mean ± standard deviation.

**FIGURE 2:**
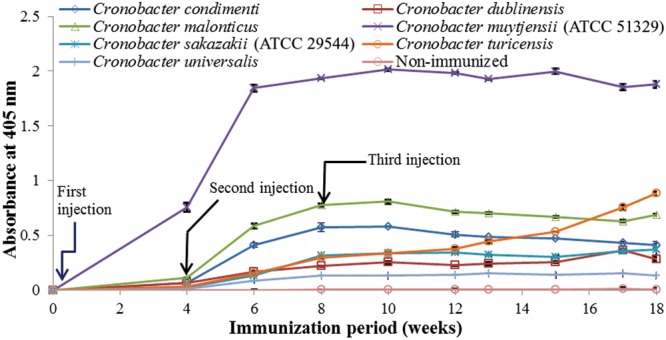
**Titers of rabbit anti-serum developed from formalin killed cells mixture against seven *Cronobacter* species.** All experiments were conducted three times, and data represent mean ± standard deviation.

**FIGURE 3:**
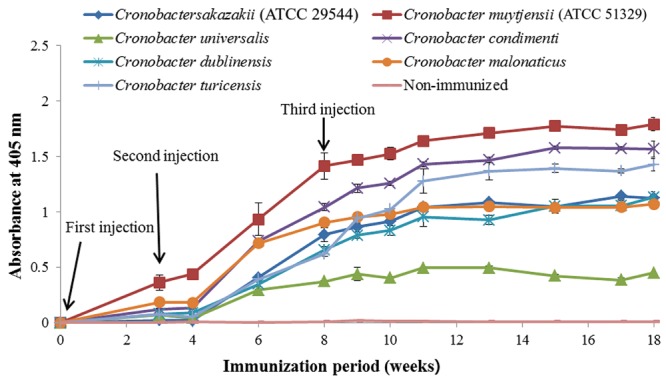
**Titers of rabbit anti-serum developed from sonicated cell protein mixture against seven *Cronobacter* species.** All experiments were conducted three times, and data represent mean ± standard deviation.

**FIGURE 4:**
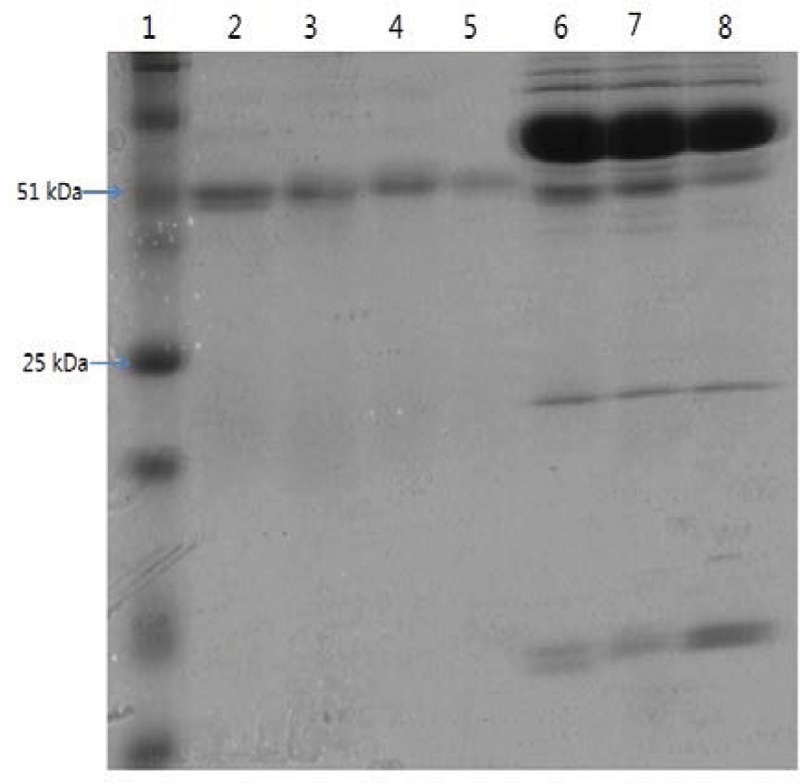
**Sodium dodecyl sulfate-polyacrylamide gel electrophoresis of developed rabbit anti-*Cronobacter* IgG and anti-serum.** Lane 1: standard protein marker, lane 2: commercial rabbit IgG, lane 3: anti-*Cronobacter* IgG developed from sonicated cell protein mixture of *Cronobacter* spp., lane 4: anti-*Cronobacter* IgG developed from formalin killed cells mixture of *Cronobacter* spp., lane 5: anti-*Cronobacter* IgG developed from lipopolysaccharide mixture of *Cronobacter* spp., lane 6: anti-serum developed from sonicated cell protein mixture of *Cronobacter* spp., lane 7: anti-serum developed from formalin killed cells mixture of *Cronobacter* spp., lane 8: anti-serum developed from lipopolysaccharide mixture of *Cronobacter* spp.

As shown in **Figure 1**, anti-serum containing anti-*Cronobacter* IgG developed by the LPS mixture showed no titer for any *Cronobacter* species. **Figure 1** provides the fact that LPS antigen of *Cronobacter* is not appropriate for the production of anti*-Cronobacter* antibody. For antibodies developed using FKC and SCP mixtures of *Cronobacter* spp., titers of the developed rabbit anti*-Cronobacter* serum containing IgG were determined by INC-ELISA after 18 weeks of immunization, as shown in **Figures 2** and **3**. Titers of the developed anti-*Cronobacter* seru containing IgG increased after the first injection as well as gradually increased after the second and third injections. Titers of anti-*Cronobacter* serum containing IgG developed from the FKC mixture of *Cronobacter* spp. (**Figure 2**) were in the following order: *C. muytjensii, C. turicensis, C. malonaticus, C. condimenti, C. sakazakii, C. dublinensis*, and *C. universalis*. Comparatively, anti-*Cronobacter* serum containing IgG developed from an SCP mixture of the seven *Cronobacter* spp. showed higher titers against the seven *Cronobacter* spp. (**Figure 3**), which were in the following order: *C. muytjensii, C. condimenti, C. turicensis, C. sakazakii, C. malonaticus, C. dublinensis*, and *C. universalis*. It is well known that an increased titer of developed antibody results in effective performance during immunoassay. The developed and purified anti-*Cronobacter* IgG showed very high purity as compared to the commercial rabbit IgG (**Figure 4**). Developed antibody as well as commercial rabbit IgG (lanes 2, 3, 4, and 5) showed a strong band around 51 kDa and a light band around 25 kDa, respectively. These results confirm that our developed rabbit anti-*Cronobacter* IgG with high purity is comparable to commercial rabbit IgG.

### INC-ELISA for Detection of *Cronobacter* spp. Using Developed Anti-*Cronobacter* IgG

An INC-ELISA method was developed to detect the seven *Cronobacter* spp. using rabbit anti-*Cronobacter* IgG developed from the aforementioned SCP mixture. The developed INC-ELISA method showed excellent sensitivity toward *Cronobacter* spp., including *C. condimenti, C. dublinensis, C. malonaticus, C. muytjensii, C. sakazakii, C. turicensis*, and *C. universalis*, confirming its ability to detect all seven *Cronobacter* spp. in a single test (**Table 1**). Furthermore, the developed INC-ELISA was highly sensitive to all seven different *Cronobacter* spp. with a detection limit ranging from (5.6 ± 0.30) × 10^3^ to (2.1 ± 0.01) × 10^5^ CFU/mL (**Table 1**).

### Specificity of Developed INC-ELISA

According to **Table 2**, the developed INC-ELISA showed no cross-reactivity with any of the other tested bacterial genera. The recognition difference between targeted bacteria (positive value; *P* value) and non-targeted bacteria (negative value; *N* value) is an important parameter affecting the results of the immunoassay. In the present study, non-targeted bacteria (non-*Cronobacter*) showed a *P*/*N* value of <2 (**Table 2**), which is considered as a negative result, whereas targeted strains showed a *P/N* value of >2, which is considered as a positive result (**Table 1**). In support of this, the *P/N* value for *F. pulveris* was around 1.942 (**Table 2**) where a *P/N* value >2 is considered as a positive result. Compared to several other methods, our newly developed INC-ELISA based on rabbit anti-*Cronobacter* IgG is less expensive and easier to perform since it does not require expensive reagents or probes and has an assay time of only 36 h comprising 8 h of pre-enrichment, 8 h of enrichment, and 20 h of INC-ELISA. During assay development, accuracy of the rapid detection method was confirmed by standard plate colony counting techniques. Moreover, the developed rapid assay is accurate enough to avoid repeated colony counting steps to save detection time.

**Table 2:** Cross-reactivity of the developed indirect non-competitive enzyme-linked immunosorbent assay with non-*Cronobacter* species in pure culture.

Species	Result	Positive/Negative values
*Escherichia coli*	-	1.002
*Salmonella* Enterica	-	1.109
*Salmonella* Typhimurium	-	1.603
*Bacillus cereus*	-	1.076
*Citrobacter freundi*	-	1.594
*Buttiauxella noackiae*	-	1.067
*Franconibacter pulveris*	±	1.942
*Franconibacter helveticus*	-	1.010

### Detection of *Cronobacter* spp. in Spiked PIF Samples

To detect small amounts of *Cronobacter* spp. cells using the developed method, PIF samples were separately spiked with each of the seven *Cronobacter* spp. at low concentrations (<10 cells/25 g). All PIF samples spiked separately with *C. condimenti, C. dublinesis, C. malonaticus, C. muytjensii, C. sakazakii, C. turicensis*, and *C. universalis* showed the presence of *Cronobacter* spp. in both the INC-ELISA and USFDA/KFDA methods, whereas unspiked samples showed the absence of *Cronobacter* spp. Specific enrichment was carried out in EE broth as per KFDA standard methods (Korea Food and Drug Administration [KFDA], 2014). In the present study, the detection limit of the developed INC-ELISA against *Cronobacter* spp. was found to be <10 cells/25 g of PIF after 16 h of enrichment (**Table 3**).

**Table 3:** Food trial for detection of *Cronobacter* and indirect non-*Cronobacter* species in artificially contaminated PIF using the developed non-competitive enzyme-linked immunosorbent assay.

Strains used for inoculation	Spiked level (CFU/25 g)	Results				
		Developed INC-ELISA	USFDA/KFDA	Colony counting on VRBG agar plate after 8 h enrichment	
**Control**	**0**	**-**	**-**	**CFU/mL**	**Log CFU/mL**
*Cronobacter condimenti*	<10	+	+	1.9 × 10^5^	5.27
*Cronobacter dublinensis*	<10	+	+	1.7 × 10^5^	5.23
*Cronobacter malonaticus*	<10	+	+	1.9 × 10^5^	5.27
*Cronobacter muytjensii* (ATCC 51329)	<10	+	+	2.9 × 10^5^	5.46
*Cronobacter sakazakii* (ATCC 29544)	<10	+	+	2.4 × 10^5^	5.38
*Cronobacter turicensis*	<10	+	+	1.5 × 10^5^	5.17
*Cronobacter universalis*	<10	+	+	1.1 × 10^5^	5.04

*+: *Cronobacter* spp. detected; -: *Cronobacter* spp. not detected.*

Further, to confirm the results obtained using the developed antibody and INC-ELISA for *Cronobacter* detection, conventional standard plate count technique using the spreading method on NA and VRBG agar plates was carried out. The PIF samples artificially contaminated with each *Cronobacter* spp. separately (<10 cells/25 g) after 8 h of pre-enrichment and 8 h of enrichment in EE broth showed similar bacterial counts for each *Cronobacter* spp. in the range of 1.1 × 10^5^ – 2.9 × 10^5^ CFU/mL on specific VRBG agar plates (**Table 3**). These results can give an idea to reconfirm the detection limit obtained in pure culture of *Cronobacter* spp. using the developed antibody and INC-ELISA assay (**Table 1**).

## Discussion

In this study, an INC-ELISA method based on our laboratory-produced anti-*Cronobacter* IgG was developed and applied for the detection of seven *Cronobacter* spp. in PIF. This study employed three types of immunogens (LPS, FKC, and SCP) to produce three types of antibodies in rabbit for the detection of seven *Cronobacter* spp. As recommended by the Canadian Council on Animal Care [CCAC] (2002), a second injection of immunogen should be performed 3 to 6 weeks after the first immunization. Therefore, in the present work, two immunogen injections were made after the first injection at an interval of 4 weeks during the immunization period. As a result, IgG developed by LPS immunogen showed no titer for any *Cronobacter* spp. These results agree with previous findings of Luk and Lindberg (1991) and Jaradat et al. (2011), who were unable to obtain stable antibodies against LPS from *Cronobacter* and *Salmonella* due to the structure and composition of LPS obtained from bacterial cells of *Cronobacter* and *Salmonella*.

Previously, Hochel and Skvor (2009) developed a serotype-specific polyclonal antibody against *E. sakazakii* (*Cronobacter* spp.) capable of detecting 13 *E. sakazakii* spp. from different isolations. Compared to the results of Hochel and Skvor (2009), the developed rabbit anti-*Cronobacter* IgG in this study showed a higher titer, purity, and specificity, which makes it suitable for developing a fast and simple immunoassay method for the detection of *Cronobacter* spp. Therefore, anti-*Cronobacter* IgG developed from an SCP mixture of the seven *Cronobacter* spp. was chosen to develop an INC-ELISA method for genus-specific detection of *Cronobacter* spp. Similarly, Liang et al. (2001) developed a monoclonal antibody for the detection of *Bartonella* spp. capable of specifically detecting all tested species of *Bartonella*.

In addition, Huang et al. (2013) designed two pairs of primers based on *gyr*B sequences for specific identification of *C. sakazakii* and *C. dublinensis*. Although these PCR-based methods can be used to rapidly analyze *Cronobacter* spp., their strict requirements in terms of technique, equipment, and probes limit their application (Ruan et al., 2013). Further, Zhang et al. (2010) reported a cross-priming amplification method combined with immunoblotting analysis for genus-specific detection of *Cronobacter* spp. under isothermal conditions.

Similarly, we developed a polyclonal antibody for *C. muytjensii* as well as fluorescence-based liposome immunoassay for the detection of *C. muytjensii* in our previous study (Song et al., 2015). In this study, the developed antibody was also able to detect other strains of *C. muytjensii* such as *C. muytjensii* ATCC 51329 and *C. muytjensii* CDC3523–75 (Song et al., 2015). Furthermore, as shown in **Table 2**, *F. pulveris* (LMG 24057) showed slight cross-reactivity with INC-ELISA at a high bacterial concentration (10^8^ CFU/mL), which might be due to close resemblance between multi-locus sequence typing loci for *Cronobacter* genus members and *Franconibacter* genera (previously recognized in *Enterobacter* genus) (Forsythe et al., 2014). Recently, Xu et al. (2014) developed a polyclonal antibody for *Cronobacter* spp. by using heat-killed antigen preparation and evaluated its cross-reactivity against various *Cronobacter* strains of the same species. Their results confirmed cross-reactivity for all *Cronobacter* strains of the same species based on positive signals. The detection limit was observed in the range of 10^4^–10^5^ CFU/mL (Xu et al., 2014). Therefore, it can be hypothesized that the developed antibody and assay in the present study could be applicable for the detection of several *Cronobacter* strains of similar species (Xu et al., 2014).

The above findings confirm that the developed assay method in this study can detect *Cronobacter* spp. in PIF samples with high sensitivity within 36 h. Similarly, Blazkova et al. (2011) developed a method using an immunochromatographic strip for the detection of *Cronobacter* spp. with a detection limit less than 10 cells/10 g of PIF. In the present study, all PIF samples artificially contaminated with *Cronobacter* spp. were positively detected using the developed genus-specific anti-*Cronobacter* IgG antibody in 25 g of PIF after 16 h of sample pre-treatment (8 h of pre-enrichment and 8 h of enrichment) and 20 h of assay time. In contrast, detection and confirmation of this pathogen by the USFDA culture-based method takes up to 3–5 days.

However, the developed method has limited differentiation ability for detection of each *Cronobacter* spp., which must be overcome to validate its practical and industrial usefulness. Hence, development of additional immunoassays based on the produced *Cronobacter* genus-specific antibody multiplexing format such as ELISA are in progress in our laboratory for the purpose of developing a novel assay method with reduced detection time, high sensitivity, and multiplexing detection ability.

In summary, this study focused on the development of a simple, quick, and sensitive genus-specific detection assay for *Cronobacter* spp. using rabbit anti-*Cronobacter* IgG. For this purpose, three immunogens, LPS mixture of *Cronobacter* spp., FKC mixture of *Cronobacter* spp., and SCP mixture of *Cronobacter* spp., were prepared and used to develop antibodies against *Cronobacter* spp. The newly developed rabbit anti-*Cronobacter* IgG was purified using caprylic acid and ammonium sulfate precipitation from anti-sera and showed high purity similar to the commercial rabbit IgG. The rabbit anti-*Cronobacter* IgG developed from the SCP mixture was used to develop a new INC-ELISA method for the detection of *Cronobacter* spp. The developed INC-ELISA showed excellent reactivity with all seven *Cronobacter* spp., excluding other non-*Cronobacter* foodborne pathogens. These results confirm that the developed INC-ELISA is highly sensitive, efficient, and rapid for the detection of *Cronobacter* spp. with a markedly reduced total detection time (from 5 days to 36 h), costs, and handling procedure. This is the first report of a genus-specific antibody and immunoassay method for the detection of *Cronobacter* spp. with the goal of reducing risk of *Cronobacter* spp. contamination in food.

## Author Contributions

XS, GL, and SP performed experiments and drafted manuscript; SS contributed interpretation, analyzed data, and wrote paper, MK contributed for conception, designed experiment, analyzed data, and provided technical support.

## Conflict of Interest Statement

The authors declare that the research was conducted in the absence of any commercial or financial relationships that could be construed as a potential conflict of interest.
